# Total Hip Arthroplasty for Rapidly Destructive Coxarthrosis in a Patient with Severe Platelet Deficiency due to Liver Cirrhosis and Immune Thrombocytopenic Purpura

**DOI:** 10.1155/2015/469879

**Published:** 2015-05-03

**Authors:** Shunpei Hama, Fumiaki Inori, Dai Momose, Sadahiko Konishi

**Affiliations:** ^1^Department of Orthopaedic Surgery, Osaka General Hospital, West Railway Company, 1-2-22 Abeno-ku Matsuzaki-cho, Osaka, Osaka Prefecture 545-0053, Japan; ^2^Department of Hematology, Osaka General Hospital, West Railway Company, 1-2-22 Abeno-ku Matsuzaki-cho, Osaka, Osaka Prefecture 545-0053, Japan

## Abstract

Rapidly destructive coxarthrosis (RDC) causes rapid and extreme destruction of the hip joint, which was reported by Postel and Kerboull. RDC is commonly unilateral and occurs mostly in elderly women. Immune thrombocytopenic purpura (ITP) is characterized by a low platelet count that is the result of both immune-mediated platelet destruction and suppression of platelet production. In patients with ITP undergoing surgery, bleeding associated with a low preoperative platelet count can lead to unsuccessful outcomes. To the best of our knowledge, there has been only one report describing total hip arthroplasty (THA) for patients with ITP and there have been no reports of THA for RDC with a very low platelet count due to liver cirrhosis (LC) and ITP. We report the case of a patient who had right RDC and a very low platelet count due to LC and ITP in whom THA was successfully performed. Furthermore, this case was also unique in that her platelet count increased after THA. THA for right RDC might resolve ITP by relieving inflammation of the right hip since her platelet count recovered after THA.

## 1. Introduction

We report a case of successful total hip arthroplasty (THA) in a patient with severe platelet deficiency due to liver cirrhosis (LC) and immune thrombocytopenic purpura (ITP). A 70-year-old woman with LC and ITP had right groin pain due to rapidly destructive coxarthrosis (RDC). It was possible to perform THA for her right RDC with adequate perioperative management, including control of the platelet count. To the best of our knowledge, there has been only one case report of THA for a patient with ITP and there have been no reports of THA for RDC with a very low platelet count due to liver cirrhosis (LC) and ITP. Furthermore, this case is unique in that the ITP improved after THA for RDC.

## 2. Case Report

**Figure 1:**
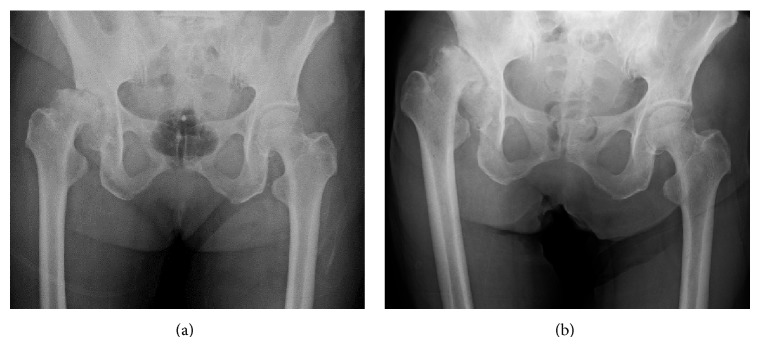
(a) A radiograph 2 months before admission to our hospital. (b) A radiograph 1 month after admission to our hospital. (a) and (b) show rapid hip joint destruction.

**Figure 2:**
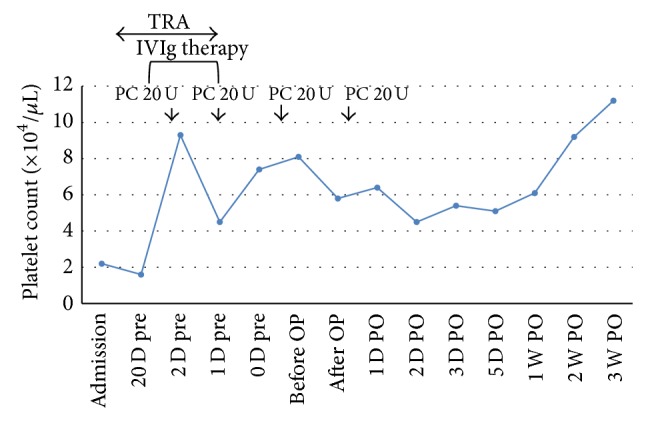
The platelet count is controlled and increased to 81000/*μ*L just before the operation. An additional 20 units of platelets are transfused after the operation. TRA: thrombopoietin receptor agonist; PC: platelet concentrate; IVIg: intravenous immunoglobulin; U: unit; Pre: preoperation; OP: operation; PO: postoperation.

**Figure 3:**
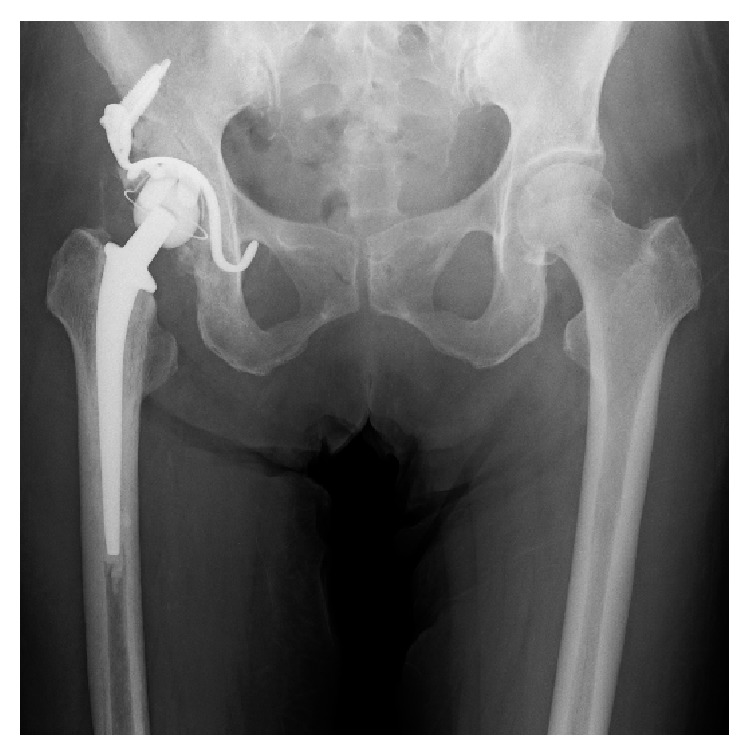
The femoral head is transplanted as a bone graft to the bone defect of the acetabulum, a KT plate is placed on the acetabulum, and cemented THA is performed.

A 70-year-old woman was admitted to the Department of Gastroenterology and Hematology of her previous hospital with the diagnosis of LC after hepatitis C and esophageal varices. To make matters worse, right groin pain, which had developed before admission, increased, and she was diagnosed as having RDC. Because her platelet count decreased to less than 10,000/*μ*L from a normal range of 50,000–80,000/*μ*L, she was referred and admitted to the Department of Hematology of our hospital, and the diagnosis of ITP was made. At the time of admission, her right groin pain was very severe, and she could not move her leg. Radiographs revealed strikingly rapid hip joint destruction (Figure 1), and her platelet count was 22,000/*μ*L. Other laboratory data are shown in Table 1. Fortunately, her LC was Child's B, so it was decided to plan THA after controlling her platelet deficiency. From 3 weeks before the operation, thrombopoietin receptor agonist injections, from 1 *μ*g/kg to 5 *μ*g/kg, increasing 2 *μ*g/kg every week, were started. In addition, 20 g/400 mL of kenketsu glovenin-I were injected for 5 days from 6 days before the operation. Twenty units of platelets were transfused 3 times, 2 days before the surgery, the day before the surgery, and immediately before the surgery. Her platelet count finally increased to 81,000/*μ*L just before the surgery (Figure 2), after which THA could be performed under general anesthesia. In order to avoid the risk of vascular damage, a posterior approach was used [1]. To decrease the amount of blood loss as much as possible, a cemented implant was chosen for both the acetabular side (E-1 acetabular liner (Biomet, Warsaw, IN)) and the femoral side (PHYSIO-HIP SYSTEM type 6 (Kyocera, Kyoto, Japan)). To reconstruct the large acetabular defect, a KT plate (Kyocera) and autograft of the femoral head were used (Figure 3). The operating time was 173 minutes, and the blood loss was 2123 mL. An additional 20 units of platelets were transfused postoperatively, and no hemostatic complications occurred. Six weeks after the operation, she could walk at full weight-bearing, and her platelet count recovered over 50,000/*μ*L. The patient was discharged with T-cane assisted walking 8 weeks after the operation. Six months after the operation, her platelet count was 57,000/*μ*L, and she could walk full weight-bearing with continued T-cane assistance. Her JOA HIP score improved from 0 points from before the operation to 69 points 6 months after THA.

## 3. Discussion

RDC causes rapid and extreme destruction of the hip joint, which was reported by Postel and Kerboull [2]. In RDC, hip joint destruction occurs rapidly within 6 to 12 months. RDC is commonly unilateral and occurs mostly in elderly women [3]. To the best of our knowledge, there have been no reports of THA for RDC with a very low platelet count due to LC and ITP. Cohen et al. presented a controlled retrospective study on the safety and outcome of THA and total knee arthroplasty (TKA) in cirrhotic patients. They reported that perioperative complications, liver decompensation, and mortality were not significantly different in 14 cirrhotic patients compared with 42 age- and sex-matched controls, and primary elective THA appeared safe in patients with Child's A and B cirrhosis [4]. The present case had Child's B cirrhosis; so it was decided to proceed with surgery.

ITP is characterized by a low platelet count that is the result of both immune-mediated platelet destruction and suppression of platelet production [5, 6]. In patients with ITP undergoing surgery, bleeding associated with a low preoperative platelet count can lead to unsuccessful outcomes. When the preoperative platelet count is less than 50,000/*μ*L, intensive perioperative management is needed to avoid platelet depletion [7, 8]. There are several methods to elevate the preoperative platelet count, and, in the present case, the patient's platelet count was increased with combination therapy involving a thrombopoietin receptor agonist, intravenous immunoglobulin, and platelet transfusions because her platelet count was less than 50,000/*μ*L. In addition, to the best of our knowledge, there has been only one report describing THA for patients with ITP. Suzuki et al. reported a case of ITP with steroid-induced avascular necrosis of the femoral head in a 68-year-old woman. Although her platelet count was 25,000/*μ*L, her platelet count increased to 94,000/*μ*L just before the operation with high-dose immunoglobulin therapy and transfusion of platelet concentrates. Therefore, THA was performed by increasing her platelet count [9].

The present case had not only ITP but also LC, such that her platelet count was very low. It was possible to perform THA since she had Child's B cirrhosis and her platelet count increased with combination therapy. Although Cohen et al. reported that there were no significant differences in blood loss between elective THA in cirrhotic patients and control patients [4], the present case lost a relatively large amount of blood during the operation. This may have been due to the fact that she had both ITP and LC.

This patient was also unique in that her platelet count increased after THA. Mizuta et al. reported a case with ITP that was preceded by ulcerative colitis (UC). In that case, UC and ITP were refractory to medical treatment, including increased dosages of steroids. However, they performed colectomy for the UC, and the platelet count recovered after colectomy. They suggested that colectomy may cure UC and ITP [10]. In the present case, it is possible that THA may have cured the RDC and ITP by relieving inflammation of the right hip, as in the UC case.

A case of thrombocytopenia due to LC and ITP who underwent THA for right RDC with perioperative management including thrombopoietin receptor agonist injections, intravenous immunoglobulin, and platelet transfusions was described. Although most patients return to lower platelet counts on cessation of thrombopoietin receptor agonists and immunoglobulin usually loses its effect 2–4 weeks after high-dose intravenous immunoglobulin therapy [11], her platelet count was over 50,000/*μ*L 6 months after the last administration of thrombopoietin receptor agonists and immunoglobulin. Therefore the patient may recover from ITP due to THA for right RDC since her platelet count increased after THA. However, longer follow-up is needed to confirm this.

## Figures and Tables

**Table 1:** Laboratory data on admission.

	Normal value	Present case
RBC (10^4^/*μ*L)	380–480	243
Hb (g/dL)	12.0–16.0	8.4
WBC (/*μ*L)	3500–8500	5400
Plt (10^4^/*μ*L)	13–32	2.2
TP (g/dL)	6.6–8.2	6.9
Alb (g/dL)	4.1–5.2	2.4
T-Bil (mg/dL)	0.2–1.2	0.9
AST (IU/L)	8–38	43
ALT (IU/L)	4–44	12
LDH (IU/L)	106–211	312
LAP (IU/L)	30–70	65
S-AMY (IU/L)	43–116	67
BUN (mg/dL)	8–23	21
Cr (mg/dL)	0.7–1.4	0.62
CRP (mg/dL)	<0.30	2.77
PT (%)	80–130	56
PT-INR	1.0	1.38
APTT (s)	25.5–37.0	40.9
Fibrinogen (mg/dL)	200–400	202
